# Union Exon Based Approach for RNA-Seq Gene Quantification: To Be or Not to Be?

**DOI:** 10.1371/journal.pone.0141910

**Published:** 2015-11-11

**Authors:** Shanrong Zhao, Li Xi, Baohong Zhang

**Affiliations:** Clinical Genetics and Bioinformatics, Pfizer Worldwide Research & Development, Cambridge, Massachusetts, 02139, United States of America; Georgia Institute of Technology, UNITED STATES

## Abstract

In recent years, RNA-seq is emerging as a powerful technology in estimation of gene and/or transcript expression, and RPKM (Reads Per Kilobase per Million reads) is widely used to represent the relative abundance of mRNAs for a gene. In general, the methods for gene quantification can be largely divided into two categories: transcript-based approach and ‘union exon’-based approach. Transcript-based approach is intrinsically more difficult because different isoforms of the gene typically have a high proportion of genomic overlap. On the other hand, ‘union exon’-based approach method is much simpler and thus widely used in RNA-seq gene quantification. Biologically, a gene is expressed in one or more transcript isoforms. Therefore, transcript-based approach is logistically more meaningful than ‘union exon’-based approach. Despite the fact that gene quantification is a fundamental task in most RNA-seq studies, however, it remains unclear whether ‘union exon’-based approach for RNA-seq gene quantification is a good practice or not. In this paper, we carried out a side-by-side comparison of ‘union exon’-based approach and transcript-based method in RNA-seq gene quantification. It was found that the gene expression levels are significantly underestimated by ‘union exon’-based approach, and the average of RPKM from ‘union exons’-based method is less than 50% of the mean expression obtained from transcript-based approach. The difference between the two approaches is primarily affected by the number of transcripts in a gene. We performed differential analysis at both gene and transcript levels, respectively, and found more insights, such as isoform switches, are gained from isoform differential analysis. The accuracy of isoform quantification would improve if the read coverage pattern and exon-exon spanning reads are taken into account and incorporated into EM (Expectation Maximization) algorithm. Our investigation discourages the use of ‘union exons’-based approach in gene quantification despite its simplicity.

## Introduction

In recent years, RNA-sequencing (RNA-seq) is emerging as a powerful technology in transcriptome profiling, and this technique allows an in-depth look into the transcriptome [1–3]. In our previous study, we have investigated T cell activation using both RNA-seq and microarray in parallel. Our side by side comparison has demonstrated RNA-seq has several advantages over microarrays [4]. RNA-seq not only avoids some of the technical limitations in a microarray experiment such as varying probe performance, and nonspecific hybridization, but also detects alternative splicing isoforms and subtle changes of splicing under different conditions. RNA-seq is becoming increasingly popular and widely used in biological research. An analysis of RNA sequencing data from 1641 samples across 43 tissues from 175 individuals in Genotype-Tissue Expression (GTEx) project has revealed the landscape of gene expression across tissues, and catalogued thousands of tissue-specific expressed genes [5–6]. These findings provide a systematic understanding of the heterogeneity among a diverse set of human tissues.

Current RNA-seq approaches use shotgun sequencing technologies such as Illumina, in which millions or even billions of short reads are generated from a randomly fragmented cDNA library. The first step and a major challenge in RNA-seq data analysis is the accurate mapping of sequencing reads to their genomic origins including the identification of splicing events. Despite of the fact that a large number of mapping algorithms have been developed for read mapping [7–10] in recent years, however, accurate alignment of RNA-seq reads remains a challenging problem mainly because of exon-exon spanning junction reads and the ambiguity of multiple-mapping reads. Nowadays, many RNA-seq alignment tools, including STAR [11], GSNAP [12], MapSplice [13], and TopHat [14], use reference transcriptomes to inform the alignment of junction reads. The advantage of using a reference transcriptome in the mapping of RNA-seq reads have been demonstrated in our previous two papers [15–16]. In fact, this has become a common practice in RNA-seq data analysis, and its necessity is becoming more evident for longer reads.

The second key step in RNA-seq data analysis is to quantify expression levels of genes, transcripts, and exons. Acquiring the transcriptome expression profile requires genomic elements (i.e. genes, transcripts, or exons) to be defined in the context of the genome. In addition to RefSeq annotation [17], there are several other public human genome annotations, including UCSC Known Genes [18], Ensembl [19] and GENCODE [20]. Characteristics of these annotations differ because of variations in annotation strategies and information sources. The choice of a gene model has a dramatic effect on both gene quantification and differential analysis, as we have demonstrated in our comprehensive evaluation [16]. In addition, Wu et al. [21] have also shown that the selection of human genome annotation results in different gene expression estimates. GENCODE [20] annotation is based upon Ensembl [19] but with improved coverage and accuracy, and used by the ENCODE consortium as well as many other projects (e.g., 1000 Genomes) as the reference gene set. In this evaluation, we therefore also chose the Gencode annotation as well.

Gene quantification and differential analysis results are dependent upon not only the choice of gene annotation [16,21], but also the computational methods for inference of transcript isoform and/or gene abundance. Since RNA-seq has become a commonplace in molecular biology laboratories, quite a number of methods have been developed for the inference of gene and isoform abundance, including RSEM [22–23], Cufflinks [24], IsoEM [25], featureCounts [26] and HTSeq [27]. In general, the methods for gene quantification can be largely divided into two categories: transcript-based approach (such as RSEM [23]) and ‘union exon’-based approach (such as featureCounts [26]). Transcript-based approach fundamentally relies upon EM algorithm to distribute reads among transcript isoforms, and this algorithm can handle reads aligning to multiple genomic loci as well as those aligning to loci for which more than one feature is annotated. However, estimating the expression of individual isoform is intrinsically more difficult because different isoforms of a gene typically have a high proportion of genomic overlap. In contrast, ‘union exon’-based counting method is much simpler. First, all overlapping exons of the same gene are merged into ‘union exons’. Then per-gene read counts are generated by intersecting all mapped reads with ‘union exons’ of the gene. A read is counted to the gene as long as it has sufficient overlap with any of ‘union exons’ or junctions. Compared with isoforms, reads can generally be assigned to genes with higher confidence. Therefore, the union exons’-based counting method is widely used in RNA-seq gene quantification though gene-level counts do not distinguish between isoforms when multiple transcripts are being expressed from the same gene under a biological condition.

Most recently, Kanitz et al. [28] carried out a systematic evaluation of the accuracy of 14 methods that have been proposed for estimating transcript isoform abundance from RNA sequencing data, and they found that many tools have good accuracy and yield better estimates of gene-level expression compared with commonly used count-based approaches, but they vary widely in memory and runtime requirements. It was also found that the quantification of gene expression is more accurate if gene expression levels are computed by cumulating the expression levels of transcript isoforms than by ignoring the transcript structures. Biologically, a gene is expressed in one or more transcript isoforms. Therefore, transcript-based approach is logistically more valid than ‘union exon’-based approach. Despite of the fact that gene quantification is a fundamental task in most RNA-seq studies, however, no systematic evaluation has been performed to assess the concordance among these two approaches in practical RNA-seq data analysis, and it remains unclear whether ‘union exon’ based approach for RNA-seq gene quantification is a good practice or not.

In this paper, we used RPKM to represent gene expression levels, and carried out a side by side comparison of ‘union exon’-based approach with transcript-based approach and evaluate the concordance between these two approaches in quantifying mRNA abundance. Furthermore, we investigated the effect of gene structure features on the difference of gene quantification by using ‘union exon’-based and transcript-based approaches. In addition, we performed differential analysis of the same dataset at both gene and transcript levels using edgeR/voom [29–31], and the differential analysis results were further compared. The limitation of isoform quantification was also explored.

One significant shortcoming of the standard RNA-seq protocol is that it loses the strand of origin information for each transcript, and accordingly, it becomes difficult to accurately determine gene expression from overlapping genes, i.e., those genes that share at least partially overlapping genomic coordinates, but are transcribed from opposite strands. Knowing the strand information of the cDNA is essential to determine from which of the overlapping genes the RNA transcript originates [32–33]. Our side by side comparison of stranded RNA-seq with non-stranded RNA-seq [34] has concluded that stranded RNA-seq provides a more accurate estimate compared with non-stranded RNA-seq. Therefore, we chose a stranded dataset to evaluate different approaches for gene quantification in this paper.

## Results and Discussions

### Reads mapping and counting

**Fig 1:**
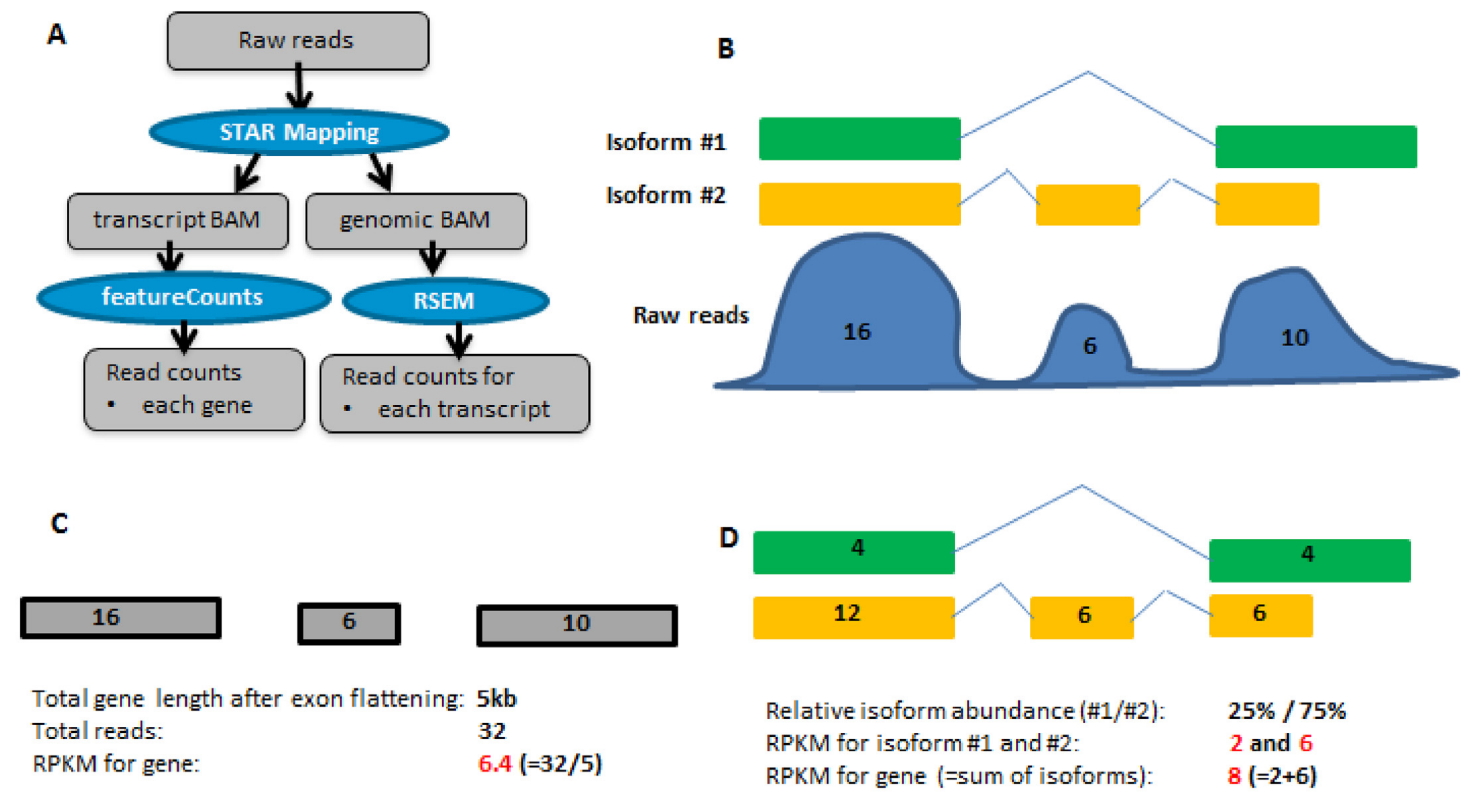
Analysis workflow and illustration of different methods for gene quantification. A) The overall mapping and counting workflow. B) A hypothetical gene, its two isoforms and read coverage profile. Assuming that the sum of mapped reads from all genes is 1 million, and each small and large exon is 1kb and 2kb long, respectively. C) featureCounts results, denoted as fc_rpkm. After exon flattening, the ‘union exons’ are 2 kb, 1 kb and 2 kb long, respectively. The calculated RPKM is 6.4. D) RSEM results, denoted as rsem_txSum_rpkm. Mapped reads are first distributed to individual isoforms, and the expressions for the two isoforms are 2 RPKM and 6 RPKM, respectively. Accordingly, the entire gene expression is 8 RPKM. Note in **Fig 1D**, the reads distributed to individual isoforms can be added up first, and then the sum of reads is divided by the length of union exons to give rise to rsem_rpkm.

The dataset and analysis protocol are detailed in the Method section, and summarized in **Fig 1A**. Briefly, the stranded RNA-seq dataset consisting of 2 UHRR (Universal Human Reference RNA) and 2 HBRR (Human Brain Reference RNA) were downloaded from Illumina’s BaseSpace. GENCODE Release 19 [20] was chosen as the reference gene set in gene and isoform quantification step in **Fig 1A**. Raw sequence reads were mapped to human genome hg19 by STAR [11] and the uniquely mapped reads were counted by RSEM [22–23] package and featureCounts [26] in the Subread package, respectively. **Table 1** reports the statistics of STAR mapping and featureCounts counting. Approximately, 90% of sequence reads can be uniquely mapped to human genome. Of those mapped reads, about 88% are in genomic exon regions that are uniquely annotated, while there are 2–2.5% of reads mapped to loci where more than one feature is annotated. Such ambiguous reads are ignored by featureCounts but counted in RSEM.

**Table 1:** The STAR mapping and featureCounts counting summaries.

Sample	STAR Mapping Summary			featureCounts Counting Summary			
	Total_reads	Unique(%)	Unmap(%)	Mapped_Reads	Gene(%)	Ambiguity(%)	No_Feature(%)
HBRR_C4	97730535	87.98	12.02	85957548	88.95	2.11	8.93
HBRR_C6	94064211	92.26	7.74	86759046	88.48	2.05	9.47
UHRR_C1	83374339	88.26	11.74	73564305	87.12	2.49	10.39
UHRR_C2	84897013	89.43	10.57	75901362	87.69	2.56	9.75

There are over 57,000 genes in GENCODE Release 19, but the majority of genes don’t have appreciable expression at all in the 4 samples. For our evaluation, we excluded the following genes.

- Originating from mitochondrion;
- Overlapping with any other gene on the same strand;
- Without appreciable expression, i.e. the read counts are less than 100 across all 4 samples;
- The combined length of ‘union exons’ is shorter than 500 bp.

The algorithm implemented in featureCounts cannot handle reads mapped to loci corresponding to more than one gene, and thus overlapping genes on the same strand are excluded. Small genes, such as microRNA and anti-sense transcripts, are discarded simply because they are too short for our evaluation. After filtering, a total of 11,634 genes are kept, and this is the gene set for downstream evaluation and analysis. The gene counts table for those 11,634 genes generated by featureCounts and the corresponding isoform counts table for 86,719 transcripts from those genes generated by RSEM are tabulated in **S1 Table**.

The total reads counted by featureCounts (denoted as fc_count) and RSEM (denoted as rsem_count) are summarized in **Table 2**. Slightly more reads are counted in featureCounts than in RSEM. To understand the ratios in **Table 2**, we need to keep two important facts in mind. First, ambiguous reads are counted by RSEM but ignored by featureCounts, whereas reads partially overlap with a gene/transcript are dropped by RSEM but counted by featureCounts. Quite often, such partially overlapping reads are intron retention reads. For those 11,634 filtered genes, all counted reads are uniquely annotated, and none of them is ambiguous. Therefore, the difference between fc_count and rsem_count approximately represents the number of intron retention reads. According to **Table 2**, about 4% of reads counted by featureCounts are intron retention reads. For “All genes”, ambiguous reads are counted in RSEM, and that’s why the calculated ratios become smaller compared with “Filtered genes”. The magnitude of ratio drop from “Filtered genes” to “All genes” approximately equals to the percentage of ambiguous reads in **Table 1**.

**Table 2:** The total number of counted reads by featureCounts and RSEM.

Reads	All genes*^1^				Filtered genes (11634)			
	HBRR_C4	HBRR_C6	UHRR_C1	UHRR_C2	HBRR_C4	HBRR_C6	UHRR_C1	UHRR_C2
**fc_count**	64024052	64658363	59538166	62146717	43404482	43704815	39448095	41261435
**rsem_count**	62857039	63494011	58624841	61238508	41642175	41967121	37811679	39553948
**Ratio** *^2^	1.019	1.018	1.016	1.015	1.042	1.041	1.043	1.043

*^1^ Those genes originated from mitochondrion are excluded.

*^2^ Ratio = fc_count/resem_count

### RPKM calculation methods

**Fig 1B** to **1D** highlight the difference between RSEM and featureCounts. The hypothetical gene has two isoforms (**Fig 1B)**. For simplicity, we assume the lengths for long and short exons are 2 kb and 1 kb, respectively, and the total number of reads is 1 million. As shown in **Fig 1C,** featureCounts first collapses overlapping exons into 3 ‘union exons’, and then calculates the RPKM by using the total length of ‘union exons’. The result is 6.2 RPKM. RSEM first distributes reads to isoforms using EM algorithm, and then isoform expressions are calculated. The gene expression is computed by cumulating the isoform expressions, and is estimated to be 8 RPKM (**Fig 1D**). In **Fig 1D**, the reads assigned to all isoforms can also be added up first, and then divide by the total length of ‘union exons’ in **Fig 1C**. In summary, there are three ways to calculate RPKM for every gene and they are denoted as follows:

1. fc_rpkm. As shown in **Fig 1C**, it is calculated from the total number of reads counted by featureCounts and the total length of ‘union exons’;
2. rsem_rpkm. Similar to fc_rpkm, but the total number of reads is the sum of reads from individual transcripts quantified by RSEM;
3. rsem_txSum_rpkm. As shown in **Fig 1D**, the RPKM of individual transcript is calculated first, and then the gene RPKM is represented as the sum of its individual transcript RPKM.

Transcript-based result rsem_txSum_rpkm is logistically more valid than ‘union exon’-based result, i.e. rsem_rpkm or fc_rpkm. Quite often, the rsem_rpkm is very close to fc_rpkm. By using these formulas, the RPKMs (i.e. fc_rpkm, rsem_rpkm, rsem_txSum_rpkm) calculated for those 11,634 genes are reported in **S2 Table**.

### Comparison of gene quantifications

**Fig 2:**
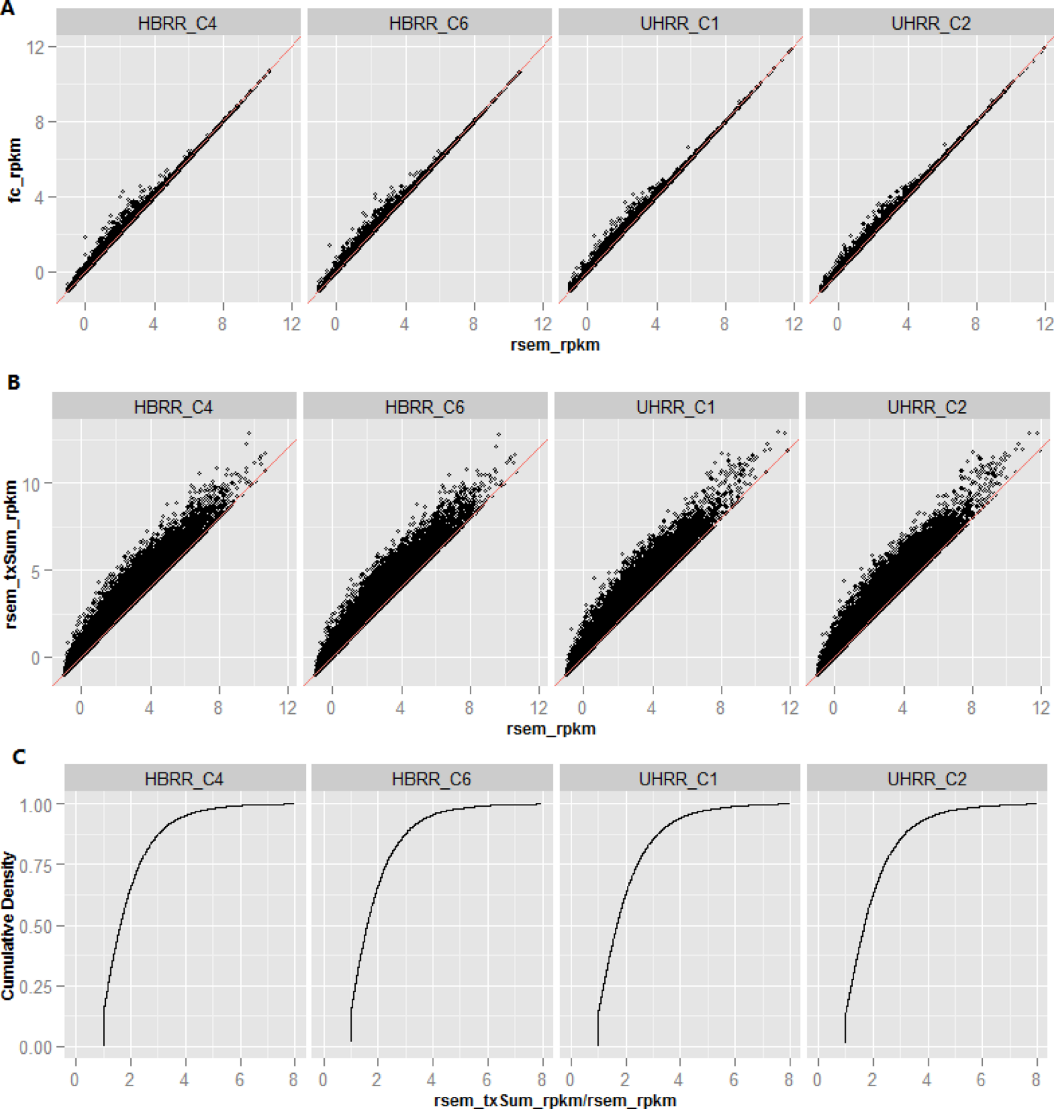
A) The scatter plot for “fc_rpkm versus rsem_rpkm”. In general, the results reported by RSEM and featureCounts are very close, and nearly identical for high expression genes. B) The scatter plot for “rsem_txSum_rpkm versus rsem_rpkm”. The difference between rsem_txSum_rpkm and rsem_rpkm is much larger than the difference between fc_rpkm and rsem_rpkm. C) The cumulative distribution of RPKM ratio (rsem_txSum_rpkm / rsem_rpkm). Note in A) and B), the x-axis and y-axis represent log2(RPKM+0.5). To avoid the division by zero, the ratio in C) is calculated from RPKM, and a background value 0.01 is added to original RPKM.

The scatter plots for “fc_rpkm vs rsem_rpkm” and “rsem_txSum_rpkm vs rsem_rpkm” are shown in **Fig 2A** and **Fig 2B**, respectively. From **Fig 2B**, the ratio of rsem_txSum_rpkm over rsem_rpkm for individual genes can be calculated, and its cumulative distribution is plotted in **Fig 2C**. Note in **Fig 2A and 2B**, the x-axis and y-axis represent log2(RPKM+0.5). For a gene with very low expression, the log2 transformation of its RPKM gives rise to a very large negative number or becomes infinite when a RPKM equals to zero. To alleviate this situation, a constant 0.5 is added to original RPKM. It is noted the ratio in **Fig 2C** is calculated from original RPKMs, not from log2 transformed values. To avoid the division by zero, a background RPKM 0.01 is added to original RPKM prior to calculation of RPKM ratio.

**Fig 3:**
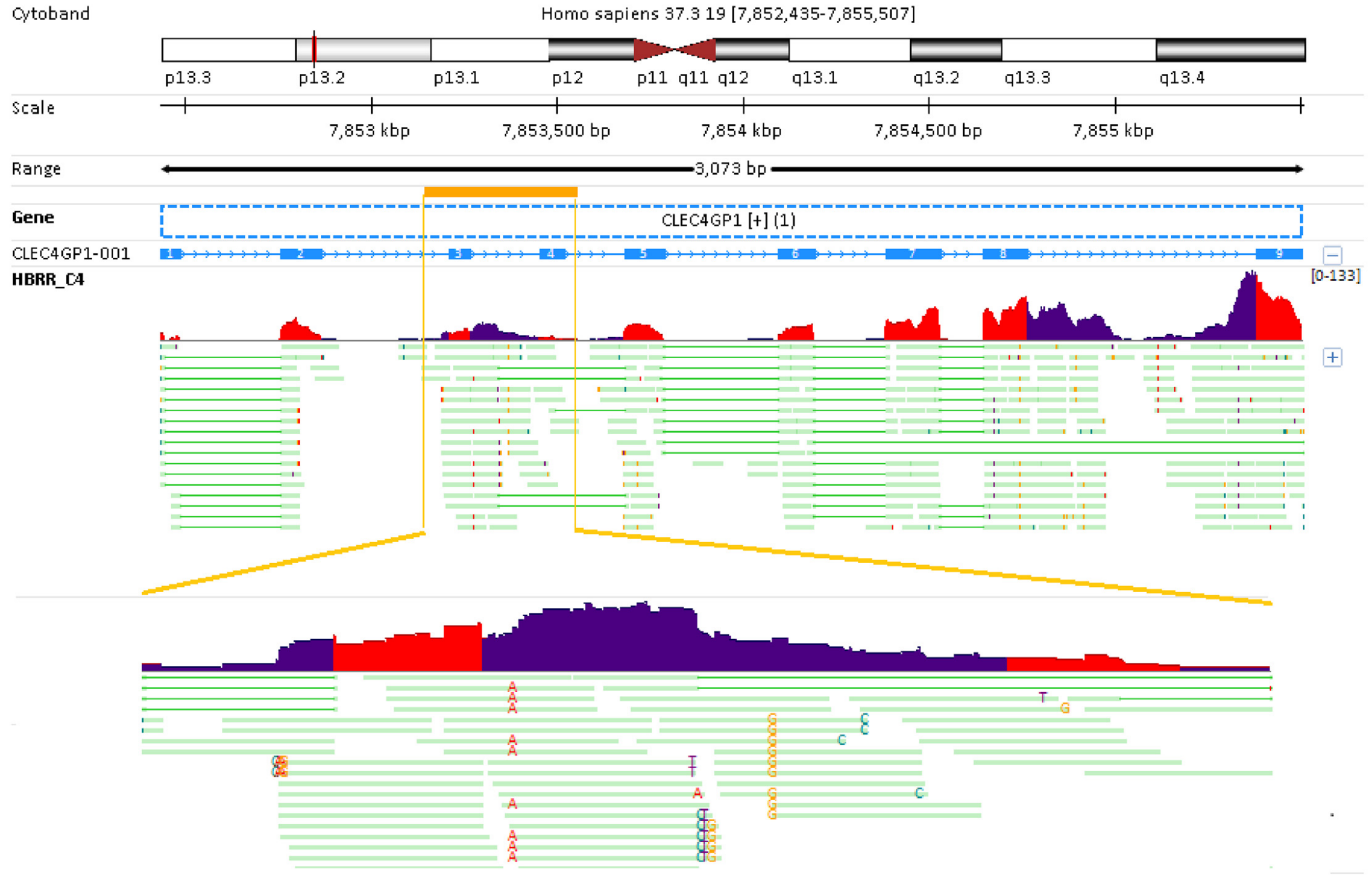
The effect of intron retention reads on gene quantification. Gene CLEC4GP1 has only 1 known transcript consisting of 9 exons. In sample HBRR_C4, many intron retention reads are mapped to gene CLEC4GP1, and counted by featureCounts. The calculated fc_rpkm (19.04) is much higher than rsem_rpkm (4.00). The exon and intron regions in coverage profile track are colored in red and dark blue, respectively. Note sample HBRR_C4 is pair-end sequenced, and only the first reads are shown in the read alignment profile.

Nearly all genes are arrayed along the diagonal line in **Fig 2A**, and this means the number of reads counted by featureCounts and RSEM have very high concordance. The gene quantification results for highly expressed genes are nearly identical. As explained above, the difference between fc_rpkm and rsem_rpkm mainly results from intron retaining reads, and the impact of intron retention reads on gene quantifications is attenuated in highly expressed genes. However, the effect of intron retention reads on low or moderately expressed genes can be more evident or dramatic. For example, in sample HBRR_C4, the calculated fc_rpkm and rsem_rpkm for gene CLEC4GP1 (Ensembl ID: ENSG00000268297.1) is 19.04 and 4.00, respectively. As illustrated in **Fig 3**, gene CLEC4GP1 is encoded at “+” strand, and has only 1 known transcript consisting of 9 exons. The exon and intron regions in read coverage track are colored in red and dark blue, respectively. As seen from the read coverage track, in addition to reads mapped to exons, there are many reads mapped to exon-intron junctions and/or intron regions which is better viewed from the read alignment profile in the zoomed genomic region around exons #3 and #4. The majority of intron retention reads in Fig **3** are counted by featureCounts but completely ignored by RSEM. That’s why fc_rpkm is much higher than rsem_rpkm.

The emergence of RNA-seq provides tremendous opportunities for researchers to detect intron retention (IR) events on a genome-wide scale. Bai et al. [35] have developed IRcall and IRclassifier to detect IR events from RNA-seq data. The methods combine together gene expression information, read coverage within an intron, and read counts (within introns, within flanking exons, supporting splice junctions, and overlapping with 5′ splice site/ 3′ splice site) to detect IR events by employing ranking strategy and classifiers. Intron retention is the most prevalent alternative splicing type in plants, and it is not uncommon in humans. How to deal with intron retention reads in gene quantification remains a debatable question. As demonstrated in **Fig 3**, intron retention reads contribute a large portion to the RPKM for gene CLEC4GP1. For those genes with large number of intron retention reads, whether to include or exclude such reads in counting step has a large impact on gene quantification results.

**Fig 4:**
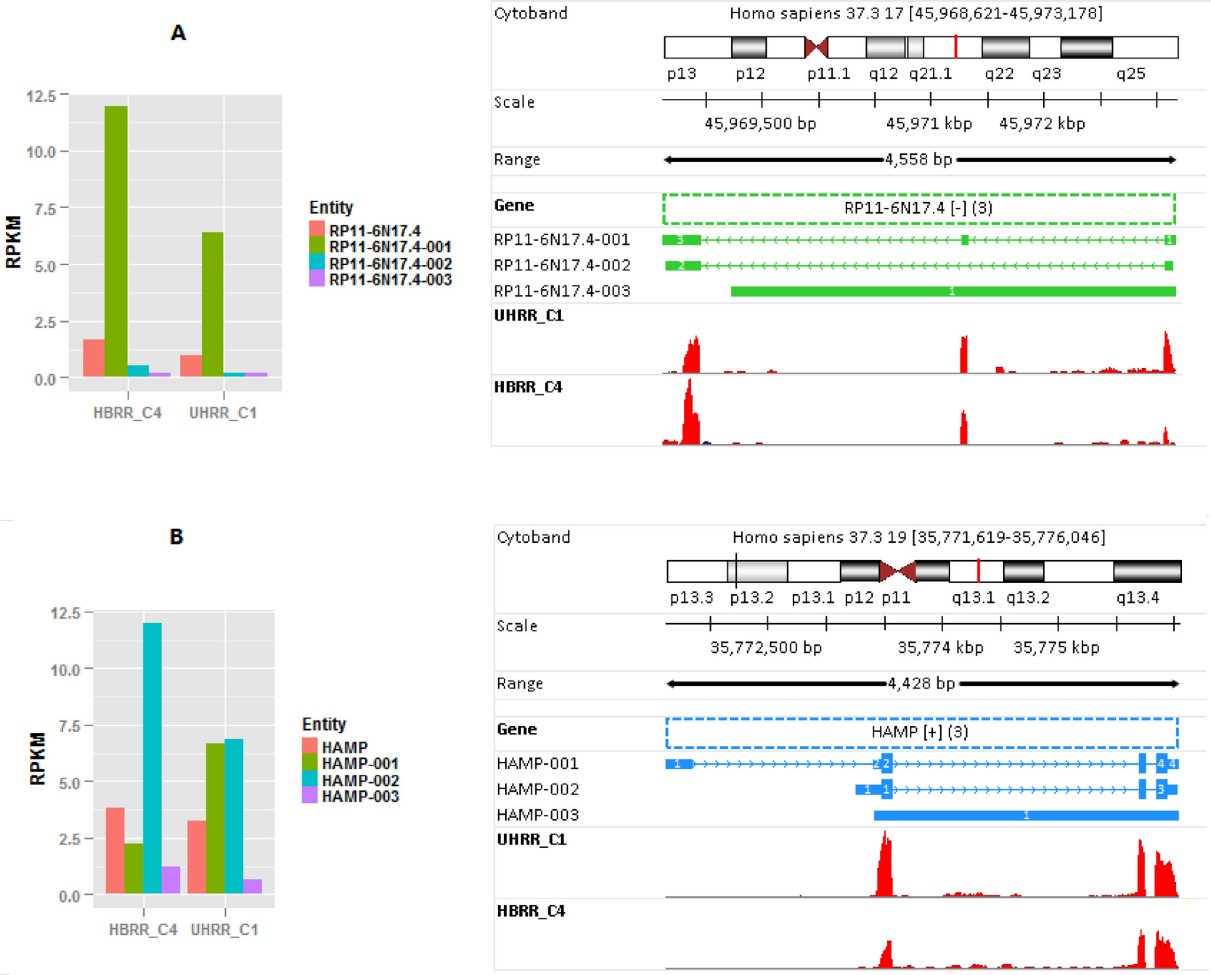
The large difference between rsem_rpkm and rsem_txSum_rpkm for genes RP11-6N17.4 and HAMP. A) The structures and expressions for gene RP11-6N17.4 and its isoforms. B) The structures and expressions for gene HAMP and its isoforms. ‘Union-exon’-based approach is incorrect for a gene that expresses multiple isoforms of varying length and the short isoforms are prevalent in expression.

Compared with **Fig 2A**, the concordance between rsem_txSum_rpkm and rsem_rpkm (see **Fig 2B**) is getting worse, even for those highly expressed genes. According to the formula, rsem_txSum_rpkm is always equal to or greater than rsem_rpkm. As mentioned earlier, rsem_txSum_rpkm is much higher than rsem_rpkm for a gene that expresses multiple isoforms of varying length and the shorter isoforms are dominant in terms of expression levels. The two genes shown in **Fig 4** clearly reveal the pitfalls for rsem_rpkm (as well as fc_rpkm). The expressions for gene RP11-6N17.4 and HAMP and their isoforms are tabulated in **Table 3**. The expressions for the two genes and their respective isoforms in samples HBRR_C4 and UHRR_C1 are plotted at left side of **Fig 4.**

**Table 3:** The rsem_rpkm and rsem_txSum_rpkm for genes RP11-6N17.4 and HAMP and their isoforms.

Measurement	RP11-6N17.4 (ENSG00000264920.1)				HAMP (ENSG00000105697.3)			
	HBRR_C4	HBRR_C6	UHRR_C1	UHRR_C2	HBRR_C4	HBRR_C6	UHRR_C1	UHRR_C2
**Ratio** *	7.70	7.32	6.93	6.88	4.06	3.47	4.33	4.47
**rsem_rpkm**	1.64	1.22	0.98	1.01	3.81	3.05	3.27	3.27
**rsem_txSum_rpkm**	12.71	8.97	6.86	7.03	15.49	10.61	14.17	14.65
**transcript_#1**	11.94	7.85	6.38	6.39	2.26	1.81	6.70	3.55
**transcript_#2**	0.54	0.86	0.24	0.38	11.98	7.31	6.84	10.46
**transcript_#3**	0.23	0.25	0.24	0.26	1.26	1.49	0.63	0.64

* Ratio = rsem_txSum_rpkm/rsem_rpkm.

Gene RP11-6N17.4 is a processed transcript, and it has 3 isoforms, consisting of 3, 2 or 1 exons (right side of **Fig 4A**), respectively. The lengths for those 3 isoforms #1/#2/#3 are 496 bp, 374 bp, and 3939 bp, respectively. Exon flattening gives rise to two ‘union exons’, and the total length of ‘union exons’ is 4558 bp. As shown in **Fig 4A**, isoform #1 has the highest expression. In sample HBRR_C4, the ratio of rsem_txSum_rpkm over rsem_rpkm is more than 7. The RPKM for isoform #1 is calculated by dividing the reads mapped to this isoform by its isoform length (i.e. 496 bp). In ‘union exon’-based approach, the same number of reads is divided by the gene length (i.e. 4558 bp). As a result, the copies of mRNA for this gene are significantly underestimated and distorted. The structures for genes HAMP and its 3 isoforms are depicted at the right side of **Fig 4B**. Its three isoforms #1/#2/#3 consist of 4, 3, or 1 exons, and their corresponding transcript lengths are 652 bp, 558 bp and 2624 bp. Two ‘union exons’ are obtained after exon flattening, and the total gene length is 3010 bp. In sample HBRR_C4, isoform #2 has the highest expression among all three isoforms. The rsem_txSum_rpkm and rsem_rpkm calculated for gene HAMP are 15.5 and 3.8, respectively (see **Table 3**). Like the gene shown in **Fig 4A**, the RPKM calculated from ‘union exon’-based approach significantly underestimates the true number of mRNA copies corresponding to gene HAMP. For those genes shown in **Fig 4**, it is incorrect to divide the read counts by the total length of ‘union exons’.

The average RPKM for those 11,634 genes in individual sample is listed in **Table 4**. The average fc_rpkm is very close to rsem_rpkm in every sample. For example, the average fc_rpkm and rsem_rpkm in sample HBRR_C4 are 19.87 and 19.81, respectively. It is also noted that the average RPKMs are very consistent across biological replicates. The difference between UHRR group and HBRR group is greater than the difference between replicates, and this is true for fc_rpkm, rsem_rpkm and rsem_txSum_rpkm. This is mainly because gene expression is very tissue specific, and the large difference between UHRR and HBRR reflects gene composition difference in these two types of samples. However, the average rsem_txSum_rpkm is much greater than fc_rpkm and/or rsem_rpkm. In sample HBRR_C4, rsem_txSum_rpkm is as high as 39.14, whereas rsem_rpkm is only 19.81. In sample UHRR_C1, the calculated rsem_txSum_rpkm and rsem_rpkm are 47.02 and 21.71, respectively, and the difference between them is even bigger. From **Table 4**, it is concluded that on average, the RPKM reported by ‘union exon’-based method is approximately 50% of the RPKM calculated by transcript-based approach, and the true gene expression levels are significantly (p-value = 0.001266) underestimated by ‘union exon’-based approach.

**Table 4:** The average RPKM for those 11634 filtered genes.

RPKM	HBRR_C4	HBRR_C6	UHRR_C1	UHRR_C2
**fc_rpkm**	19.87	19.68	21.73	21.95
**rsem_rpkm**	19.81	19.63	21.71	21.93
**rsem_txSum_rpkm**	39.14	38.32	47.02	47.51

### The RPKM ratio is gene structure dependent

**Fig 5:**
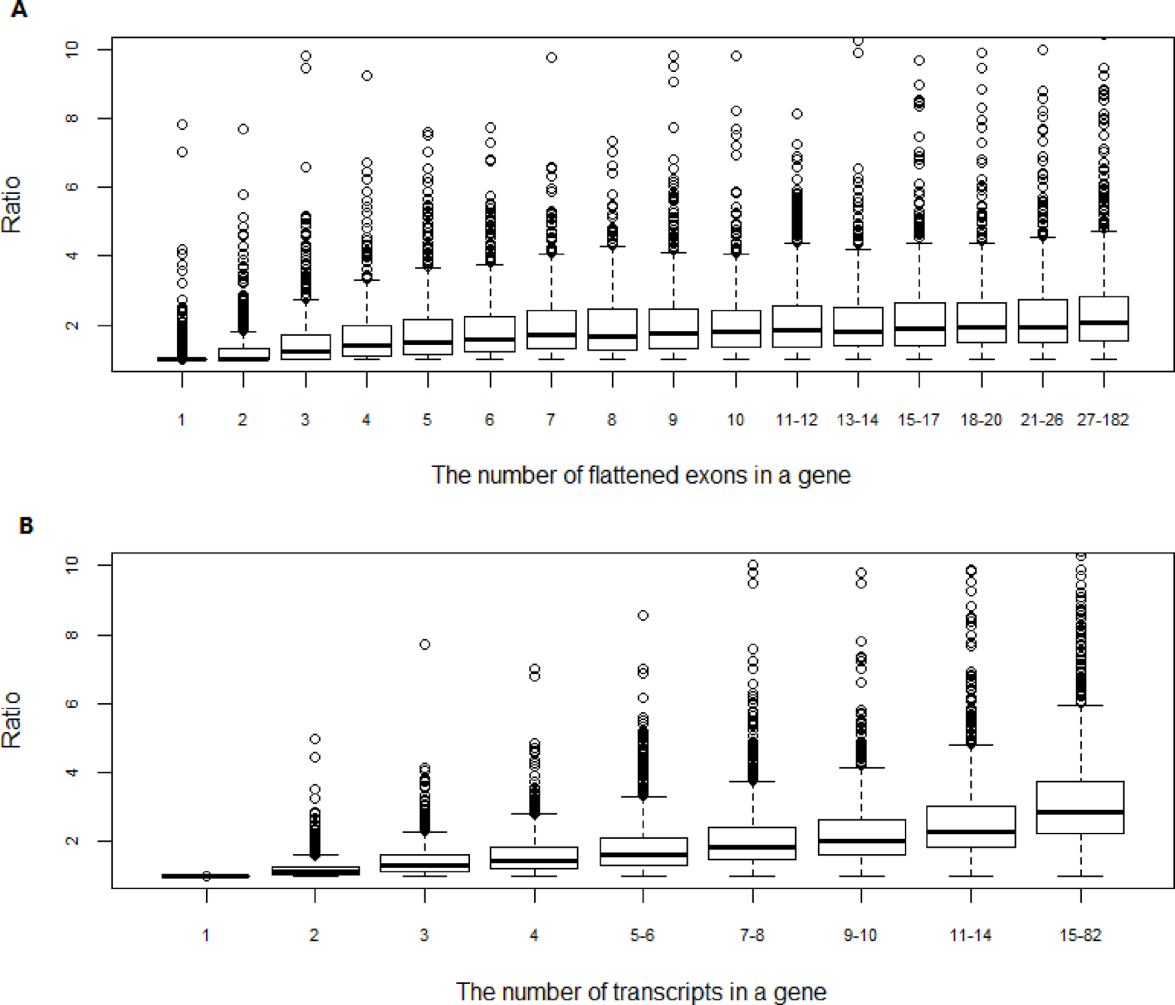
The impact of gene structural features on the difference between rsem_txSum_rpkm and rsem_rpkm. Genes are sorted according to the structural features, and then non-overlapping bins are defined containing comparable numbers of genes. A) The ratio distribution across the different bins for the number of union exons. B) The ratio distribution across the different bins for the number of transcript isoforms. Taken together, the difference between rsem_rpkm and rsem_txSum_rpkm is primarily affected by the number of transcripts in a gene.

The cumulative distribution of ratio (= rsem_txSum_rpkm/ rsem_rpkm) shown in **Fig 2C** describes the percentage of genes for a given ratio cut-off. Next, we aimed to assess the impact of gene structural features on the difference between rsem_txSum_rpkm and rsem_rpkm. Specifically, we sorted genes according to the number of ‘union exons’ of which they are composed. Likewise, we sorted genes by the number of annotated transcript isoforms. For each of the structural features, we then defined non-overlapping bins containing comparable numbers of genes. The R-style boxplots in **Fig 5** show the ratio distributions across the different bins for each of the gene structural features. If we take a look at the overall trend in **Fig 5**, the mean of RPKM ratios steadily increases with the number of ‘union exons’ (**Fig 5A**) or the number of transcript isoforms (**Fig 5B**).

For single union exon genes, the average of RPKM ratio is 1.1 (the first bin in **Fig 5A**). In comparison, for those genes consisting of 27 or more exons, the mean ratio increases to 2.46 (the last bin in **Fig 5A**). For single-transcript genes, there is no difference between rsem_txSum_rpkm and rsem_rpkm, and the ratios are always equal to 1 (the first bin in **Fig 5B**). For those genes having 15 or more transcripts, the mean ratio jumps to 3.23. The overall trends in **Fig 5A** and **Fig 5B** are to a large extent a consequence of the fact that multiple-isoform genes tend to be long and give rise to multiple-exon transcripts. Taken together (**Fig 5A** and **Fig 5B**), it is concluded that the difference between rsem_txSum_rpkm and rsem_rpkm is primarily affected by the number of transcripts in a gene. The more transcript isoforms a gene has, the larger the difference between rsem_txSum_rpkm and rsem_rpkm tends to be, and this correlation can be readily explained by the formula for rsem_txSum_rpkm. Our gene set consists of 11,634 genes, and the corresponding number of transcripts is 86,719. On average, a single gene approximately encodes 7 transcripts. Therefore, the large difference between rsem_txSum_rpkm and rsem_rpkm is expected to be commonly seen.

### Differential analysis of genes and isoforms

**Fig 6:**
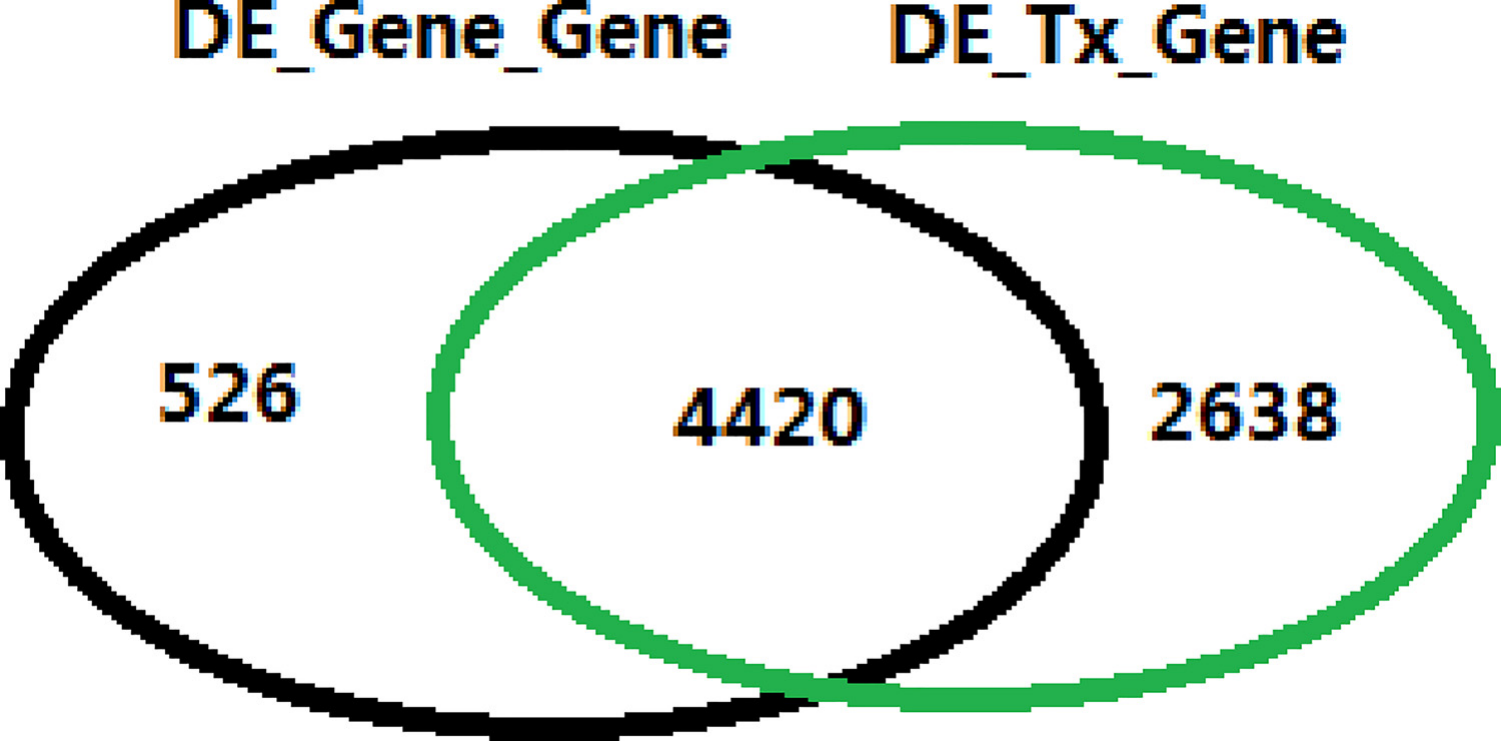
The intersection between *DE_Gene_Gene* and *DE_Tx_Gene*. Differential analysis is performed at both gene and transcript level, and *DE_Gene_Gene* contains the list of DE genes from gene level analysis. All DE isoforms are grouped by genes, and this gene list is denoted as *DE_Tx_Gene*. There are as many as 2638 genes that are not differentially expressed as a whole, but one or more of its isoforms are.

The counts table for individual transcript was generated by RSEM, and the corresponding counts table for individual gene was derived by adding up all transcript reads of the gene. The differential analyses at both gene and transcript levels were performed by edgeR/voom [29–31]. The UHRR group was compared with HRRR group. All genes or transcripts with a fold change greater than 1.5 and a Benjamini-Hochberg adjusted p-value [36] smaller than 0.05 were reported as DE (Differential Expression) genes. In general, if a gene is differentially expressed, one or more of its isoforms should be differentially expressed as well. When comparing the results between gene and isoform differential analysis, we are particularly interested in those genes that are not differentially expressed at the gene level but at the transcript level. To differentiate the two DE gene lists identified from gene and transcript analysis, the list of DE genes from gene level analysis is denoted as *DE_Gene_Gene*. All DE isoforms were grouped by genes, and this gene list is denoted as *DE_Tx_Gene*. The intersection between *DE_Gene_Gene* and *DE_Tx_Gene* is shown in **Fig 6**.

There are 4420 DE genes that are common to *DE_Gene_Gene* and *DE_Tx_Gene*, and they represent the majority of DE genes in **Fig 6**. This is readily understood since differentially expressed genes are quite often the consequence of differentially expressed isoforms. A total of 526 DE genes are unique to *DE_Gene_Gene*, and they mainly result from the difference in p-value adjustment. In multiple testing, the probability of getting a significant result simply by chance keeps going up with the number of tests. Hence, all raw p values have to be adjusted by the number of comparisons. Therefore, for a given raw p value, its adjusted p value might be still significant in gene-level testing, but no longer in transcript-level testing since much more tests are performed. On the other hand, the relationship between the variance and read counts has to be modeled first in RNA-seq differential analysis. Although in general the variance decreases with read counts, but the exact relationship at gene-level is different from the one at transcripts level. That’s why even for those single-transcript genes, the raw p values obtained from gene-level tests are not the same as those reported by transcript-level tests. Thus, those 526 DE genes unique *to DE_Gene_Gene* can be explained away by the difference in both p value adjustment and the relationship between the variance and read counts.

In the Venn diagram in **Fig 6**, as many as 2638 genes are unique to *DE_tx_Gene*. Except for 12 genes, all the rest 2626 genes consist of two or more isoforms. Surely, the difference in statistical testing discussed above can explain away some of genes in *DE_tx_Gene* list. But the majority of genes unique to *DE_tx_Gene* arise from different mechanisms. First of all, for a gene having multiple isoforms, not all isoforms are equally expressed. If the expression for a minor isoform changes significantly between two conditions, the entire gene is not necessarily differentially expressed as a whole. Secondly, it is not uncommon that the expressions increase for some isoforms, but decrease for other isoforms. Since the changes are in opposite directions, they will partially cancel out each other at the gene level. As a result, differential analysis at the gene level fails to identify the changes in isoforms. Genes *BICD2* (Ensembl ID: ENSG00000185963.9) and *ORCL* (Ensembl ID: ENSG00000122126.11) are two cases in point. The differential analysis results as well as read counts in each sample for these two genes and their isoforms are listed in **Table 5**.

**Table 5:** Differential analysis results and read counts for genes ENSG00000185963.9 and ENSG00000122126.11, and their isoforms.

Type	Ensembl ID	log2Ratio	FDR	HBRR_C4	HBRR_C6	UHRR_C1	UHRR_C2
**gene**	ENSG00000185963.9	-0.434	1.8896E-05	4438	4638	3496	3860
Transcript	ENST00000375512.3	1.509	0.0088750	643	685	2170	2319
Transcript	ENST00000356884.6	-1.685	0.0106644	3795	3953	1326	1541
**Gene**	ENSG00000122126.11	-0.544	6.7632E-07	5892	5881	4246	4593
Transcript	ENST00000357121.5	2.599	0.0383466	751	406	3775	4170
Transcript	ENST00000463271.1	1.409	0.3034412	5	2	8	14
Transcript	ENST00000486673.1	0.685	0.5620808	0	0	0	1
Transcript	ENST00000371113.4	-3.858	0.0069851	5136	5474	462	407

**Fig 7:**
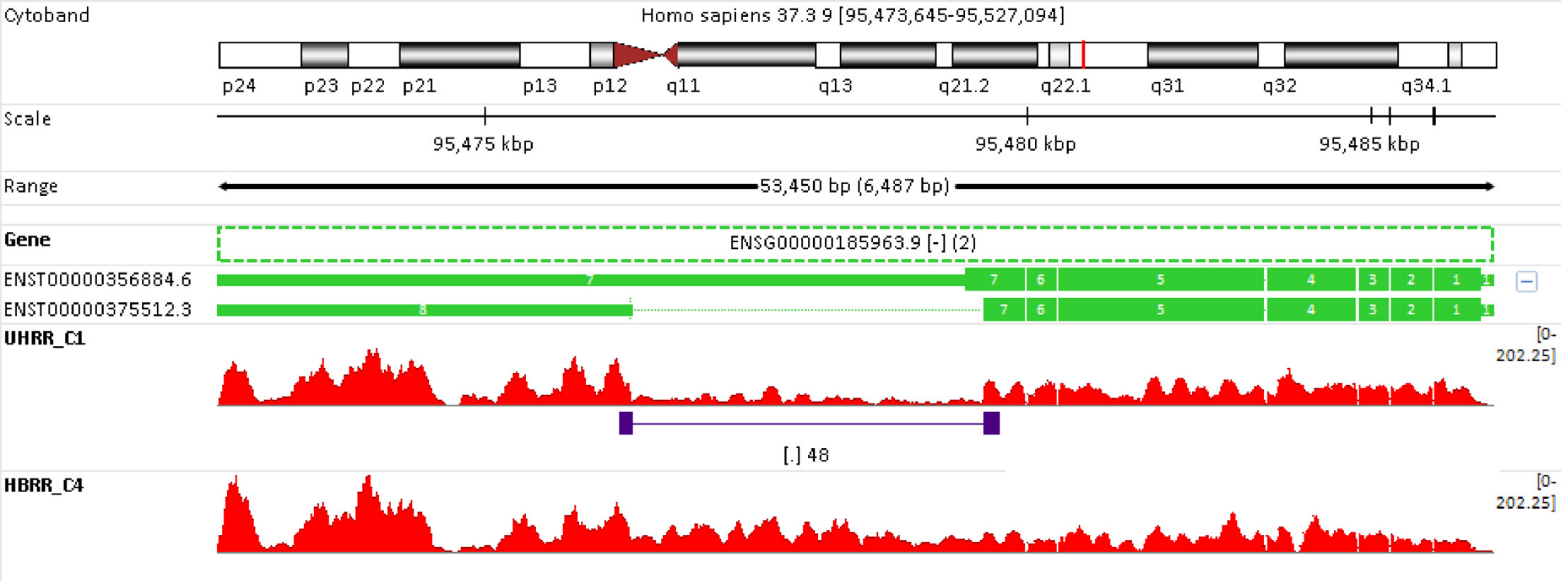
Isoform changes and switches. Gene *BICD2* (Ensembl ID: ENSG00000185963.9) consists of two very similar isoforms. At the gene level, overall, there is no much difference between sample UHRR_C1 and HBRR_C4. However, the difference is dramatic at the transcript level. For human brain sample HBRR_C4, only the long transcript ENST00000356884.6 is expressed; while in sample UHRR_C1, both isoforms are present. Note in sample UHRR_C1, there are 48 reads that span across the junction site between exons #7 and #8 (colored in indigo), and such reads can only originate from the short transcript ENST00000375512.

Gene *BICD2* (Bicaudal D Homolog 2) is encoded at “-”strand, and has two isoforms (**Fig 7**). The alternative splicing contributes to variation in Schizophrenia disease risk [37]. The two isoforms share the same first 6 exons, but differ at the rest exons. Transcript ENST00000356884.6 (termed isoform #a) has a complete exon #7, whereas transcript ENST00000375512.3 (termed isoform #b) does not. If we compare the overall gene expression in samples HBRR_C4 and UHRR_C1, there is no much difference (**Table 5** and **Fig 7**). In contrast, the isoform expression changes are obvious. In human brain sample HBRR_C4, only isoform #a is expressed, and its expression level drops by 70% in sample UHRR_C1. In addition to isoform #a, isoform #b is also presented in sample UHRR_C1. In fact, the relative abundance of isoform #b is even higher than isoform #a. The high expression of isoform #b in sample UHRR_C1 (see **Table 5**) is evidently supported by those 48 exon-exon spanning reads (colored in indigo) since they can only originate from isoform #b. Although it is evident from **Fig 7** that only isoform #a is expressed in human brain sample HBRR_C4, approximately 14% (643 out of 4438) of the reads are still assigned to isoform #b in **Table 5** by RSEM. This is mainly because when RSEM distributes reads among the isoforms, the algorithm does not take into account the read coverage pattern, such as exon-exon spanning reads. As a result, the solution from RSEM is mathematically optimal, but not biologically sound. We will come back to discuss the inaccuracy of isoform quantification later.

**Fig 8:**
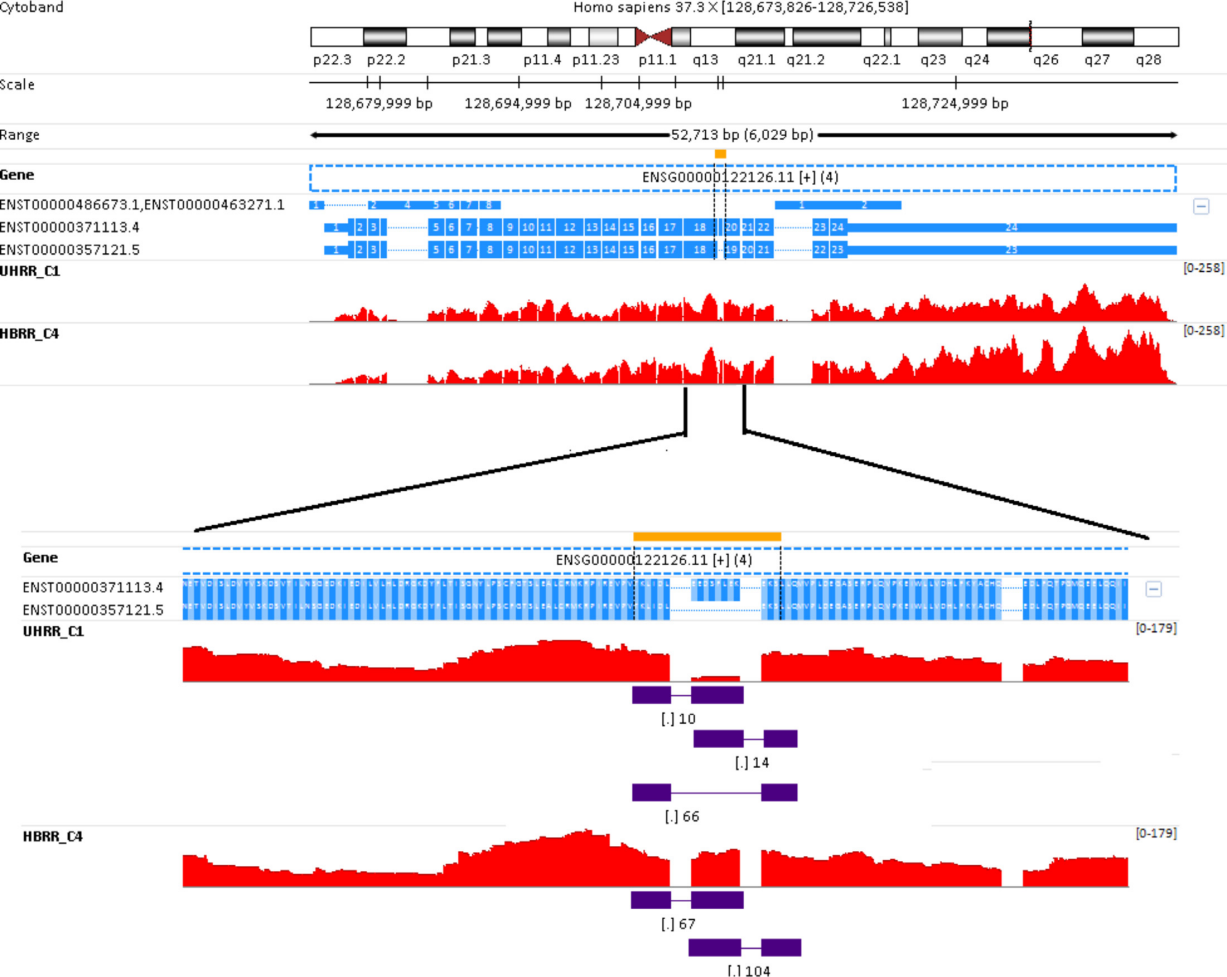
Isoform changes and switches. Gene *ORCL* (Ensembl ID: ENSG00000122126.11) has 4 isoforms. The two short isoforms don’t have appreciable expressions. The rest two long transcripts are nearly identical and differ by a single exon encoding 8 amino acids. The longer isoform ENST00000371113.4 is the only form in brain, whereas both isoforms are present in UHRR_C1. Overall, the gene *ORCL* is not differentially expressed when comparing UHRR with HBRR group, but its isoforms do, and the directions of isoform changes are opposite.

Gene *ORCL* (known as Lowe oculocerebrorenal syndrome protein) consists of 4 isoforms (**Fig 8**).The two short isoforms, i.e. ENST00000463271.1 and ENST00000486673.1, don’t have appreciable expressions (see **Table 5**). The rest two long isoforms are nearly identical and differ by a single exon encoding 8 amino acids (this region zoomed out at the bottom of **Fig 8**). The longer transcript ENST00000371113.4, termed isoform #a, is the only isoform in brain, whereas both isoforms are present in UHRR_C1. In fact, in sample UHRR_C1, the expression of ENST00000357121.5 (termed isoform #b) is much higher than isoform #a. The presence and relative abundance of isoforms are clearly indicated by those exon-exon junction reads shown at the bottom of **Fig 8**. Although only isoform #a is expressed in brain, there is still a small portion of reads (751 out of 5892) in **Table 5** are counted towards isoform #b in HBRR_C4. Like in **Fig 7**, the example in **Fig 8** again illustrates the scenario in which a gene is not differentially expressed, but its isoforms are. Quite often, the isoform changes are in opposite directions. Such dramatic isoform changes and switches are quite often masked by differential analysis at the gene level.

The significance of alternative splicing in OCRL was investigated by Choudhury et al [38]. Given its proximity to a clathrin-binding site, it is hypothesized that splicing may alter the clathrin binding properties of OCRL, and they [38] demonstrated that this is indeed the case. OCRL isoform #a binds clathrin with higher affinity than isoform #b and is significantly more enriched in clathrin-coated trafficking intermediates. In addition, they identified a second clathrin-binding site of both isoforms. Their results suggest that OCRL exists as two functional pools, one participating in clathrin-mediated trafficking events such as endocytosis and the other being much less or not involved in this process. The biological roles and functions of different isoforms from the same gene can be different, and it is important to explore isoform expression changes and their significance. We would have missed those isoform changes in **Fig 7** and **Fig 8** if we had not performed differential analysis at the transcript level.

### The inaccuracy of isoform quantification

**Fig 9:**
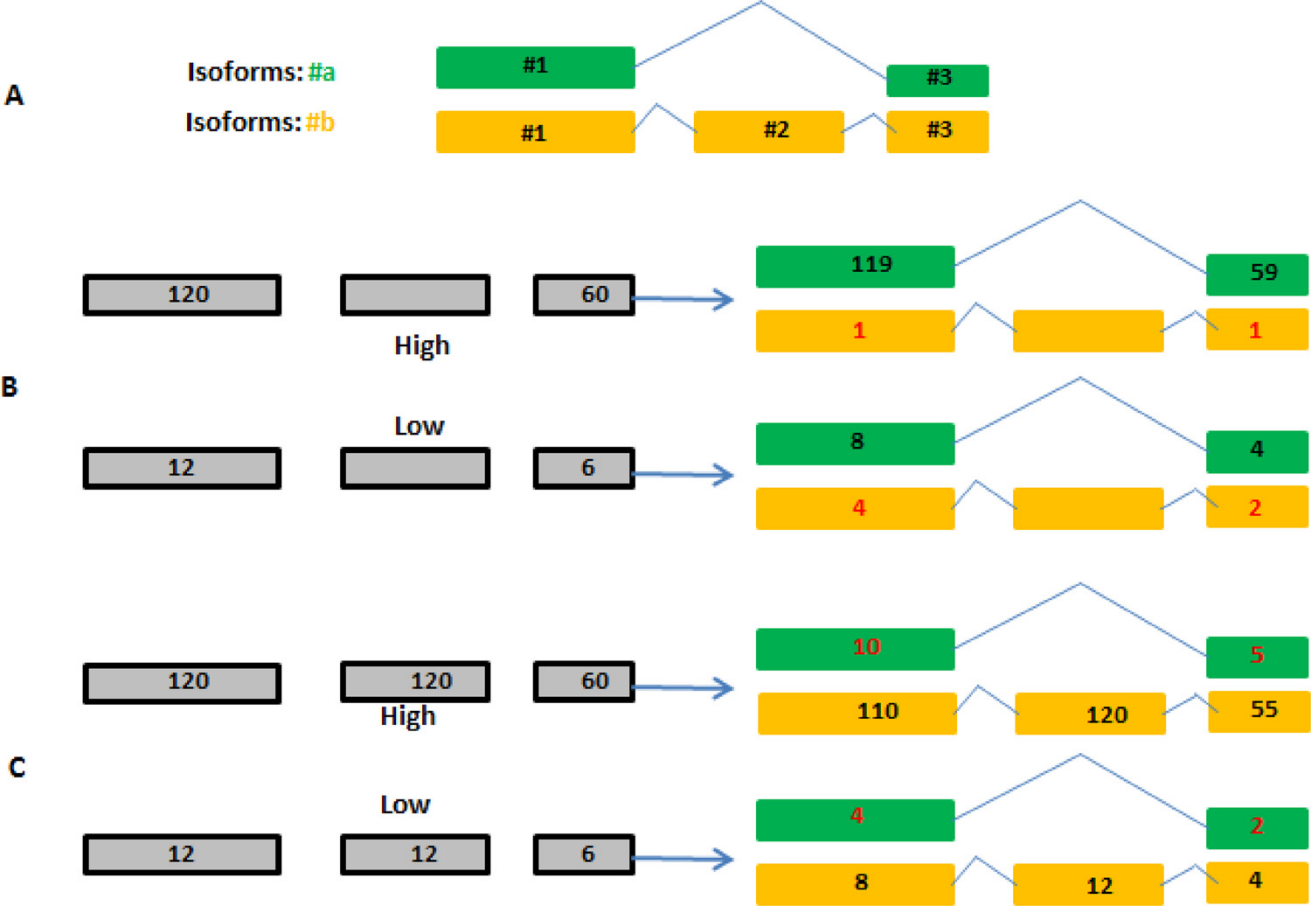
The inaccuracy of isoform quantification is influenced by the strengths of the isoforms. A) The hypothetical gene has two isoforms, termed #a and #b, respectively. Isoform #a skips exon #2, and is shorter than isoform #b. Exon #1 and #2 are twice as long as exon #3. B) Only the short isoform #a is expressed. C) Only the long isoform #b is present. In B) and C), a portion of reads are assigned to the other isoforms, and the accuracy of isoform quantification is strongly influenced by the expression level of the isoforms. More accurate isoform quantifications will be obtained if the number of exon-exon spanning reads and the read coverage pattern in isoforms are taken into account.

As we have demonstrated in **Figs 6 and 7** and **8**, more insights are gained from isoform differential analysis. However, accurate estimation of the expression levels of individual isoforms is intrinsically more difficult because of a high proportion of exon overlaps among different isoforms of the gene. In brain sample HBRR_C4, the two genes showed in **Figs 7** and **8** only express their longest isoforms. However, a small portion reads are assigned to their shorter isoforms as well (see **Table 5**). Compared with genes, reads are generally assigned to isoforms with less confidence. The accuracy of isoform quantification is crucial for differential analysis and downstream biological interpretation. To further investigate the inaccuracy of isoform quantification, we used a hypothetical gene with 2 isoforms (termed #a and #b) to illustration the pitfalls of EM algorithm (see **Fig 9**). Isoform #a skips exon #2, and is shorter than isoform #b. Exons #1 and #2 are twice as long as exon #3. For simplicity, exon-exon spanning reads are not shown in **Fig 9**, and a read is assigned to either isoform #a or #b, but not fractionally to both.

EM algorithm starts with the assumption that all isoforms are equally expressed, and splits the reads among them. It then calculates the relative abundance of individual isoforms, and then re-splits the reads according to the relative abundance. This procedure repeats again and again until it converges. In **Fig 9B**, only the short isoform #a is expressed, whereas only the long isoform #b is present in **Fig 9C**. Two panels (high and low) per isoform are shown in **Fig 9B and 9C**, respectively. In the high panel of **Fig 9B**, nearly all reads are correctly assigned to isoform #a except for 2 reads. In comparison, EM algorithm assigns one third of reads (6 out of 18) to isoform #b in the low panel. In **Fig 9C**, 5% (15 out of 300) of reads in the high panel are counted towards isoform #a, while as high as 20% of reads (6 out of 30) are assigned to isoform #a in the low panel. The isoform quantifications in **Fig 9** clearly show the accuracy is strongly influenced by the expression level of the isoforms. Highly and moderately expressed isoforms are quantified with good accuracy. On the contrary, lowly expressed isoforms are problematic. Our results are consistent with those findings in [39].

Let’s take a detail look at isoform quantification results corresponding to low expression panels in **Fig 9B and 9C**. If isoform #b in low panel of **Fig 9B** is truly expressed, we should see sufficient number of reads in exon #2, in addition to exons #1 and #3. In another word, the presence of isoform #b is not sustained by its read coverage pattern at all. Likewise, the read coverage pattern in isoform #b of **Fig 9C** also becomes problematic after 6 reads are wrongly assigned to isoform #a. As shown in **Figs 7** and **8**, exon-exon spanning reads are very informative particularly for estimating the expression of individual isoforms. Taken together, more accurate isoform quantifications will be obtained if EM algorithm can incorporate more features to inform the distributing of reads among isoforms, such as the number of exon-exon spanning reads and the isoform read coverage patterns.

Regardless of the fact that reads are generally assigned to isoforms with less reliability compared to genes, the results presented in **Figs 2** and **5** remain valid. It has been shown in **Fig 9** that the inaccuracy is not particularly biased towards either long or short isoforms. If reads are assigned to short isoforms (as in **Fig 9C**), the difference between rsem_txSum_rpkm and rsem_rpkm enlarges. Otherwise, the difference shrinks as shown in **Fig 9B**. Assuming that we have the true isoform quantifications, overall, the new scatter and distribution plots should look very similar to those plotted in **Figs 2** and **5**. The conclusion holds true that the gene quantification is significantly underestimated in ‘union exon’-based approach.

We clearly demonstrate some importance events, such as isoform switches and minor isoform changes, might be masked by gene differential analysis. Thus, quantification and differential analysis at isoform level is strongly recommended. However, we need to keep isoform quantification inaccuracy in mind, especially for those isoforms with low expression. Therefore, more false positives might be obtained from isoform differential analysis compared with gene differential analysis. A bigger hurdle for isoform differential analysis is downstream enrichment analysis, not the inaccuracy of isoform quantification. Quite often, after differential analysis is done, pathway enrichment analysis ensues. Traditionally, functional annotation and enrichment analysis have been centered on genes. Functions are frequently studied through knockout or knockdown experiments. The resulting annotations resolved at the gene level are recorded in databases such as Gene Ontology (GO) [40] and Kyoto Encyclopedia of Genes and Genomes (KEGG) pathways [41], IPA (Ingenuity Pathway Analysis) [42] and MetaCore [43]. Therefore, pathway enrichment analysis is primarily performed at the gene level. Although the recent years have witnessed an increase of the number of studies focusing on isoform specific functions, most functional annotations for proteins are still recorded at the gene level [44].

### RPKM versus TPM

In this paper, we chose RPKM to represent the relative abundance of mRNAs for a gene, and measure the concordance of gene quantifications between ‘union exon’-based approach and transcript-based method. Whether RPKM is a good measure in RNA-seq data analysis has been argued or debated for a long while [45]. Like it or not, RPKM is still widely used in RNA-seq community since its inception [1]. In this paper, we focus on the question whether exon union is a good practice or not, and RPKM is a decent measure to be used. One pitfall for RPKM is its average is not a constant, and it varies from sample to sample, as shown in **Table 4**. That’s why TPM (transcripts per million) was introduced [23] as an alternative to RPKM, and TPM fulfills the invariant average criterion. The average of TPM is always a constant as long as the number of transcripts is fixed. For instance, if the number of transcripts is 20,000, then the mean of TPM is always equal to 50 (= 10^6/20,000). If we compare TPM across samples, an implicit assumption is that the total amount of mRNAs is the same. Quite often, it is hard to justify whether this is a sound assumption or not in practical RNA-seq dataset. If this assumption does not hold true, the comparison of TPM across samples is problematic as well or even becomes pointless. On the other hand, note that within a sample the RPKM and the TPM are proportional and the RPKM can be linearly converted to the TPM by applying a scaling factor [45] that is a constant in a sample. Therefore, the conclusions drawn in our research remain the same if we choose TPM to measure gene expression.

## Conclusions

In this paper, we performed a side-by-side comparison of ‘union exon’-based approach and transcript-based method in gene expression quantification. By using RPKM to represent the relative abundance of mRNAs for a gene, it is found that the gene expression levels are significantly underestimated (p-value = 0.001266) by ‘union exon’-based approach, and that the average of RPKM from ‘union exons’-based method is approximately 50% of the mean expression obtained from transcript-based approach. The difference between the two approaches is primarily affected by the number of transcripts in a gene. The more transcripts a gene has, the larger the difference tends to be, especially when the short isoforms are the dominant transcripts. Thus, the use of transcript-based method is favored in gene quantification.

More insights are gained from differential analysis at isoform level than at gene level, and some important events such as isoform switches and minor isoform expression change are likely to be masked by gene differential analysis. However, quantifications of multiple isoforms are more complicated since different isoforms of the same gene share great part of the sequences from common exons and junctions, and the inaccuracy of isoform quantification is strongly influenced by the strength of isoforms. More accurate isoform quantifications could be obtained if EM algorithm can incorporate more features to inform the distribution of reads among isoforms, such as the number of exon-exon spanning reads and the isoform read coverage patterns

## Methods

### Stranded RNA-seq dataset and gene annotation

The stranded RNA-seq data were downloaded from Illumina’s BaseSapce [46]. The 4 samples were deeply sequenced at HiSeq 2500 platform, and the sequence read length is 75 bp. The human genome database and GENCODE annotation database were used to map and count sequence reads. The hg19 (human) genome assembly was obtained from UCSC Genome Browser, and GENCODE Release 19 was downloaded from GENCODE web site [47].

### Reads mapping and counting

The reads were mapped to the hg19 reference genome using STAR v2.4.0h [11]. The detail parameters for STAR run were “*—runThreadN 8—alignSJDBoverhangMin 1—outFilterMultimapNmax 1—quantMode TranscriptomeSAM—alignEndsType EndToEnd—outFilterMismatchNoverLmax 0*.*05—outFilterScoreMinOverLread 0*.*90—outFilterMatchNminOverLread 0*.*90—alignIntronMax 1000000—outSAMtype BAM SortedByCoordinate*”. The mapping summaries, such as the percentage of reads that were uniquely mapped and unmapped were then collected from the log files of STAR runs (see **Table 1**).

RSEM and featureCounts require the input BAM is in ‘transcriptome space’ and ‘genome space”, respectively. As shown in **Fig 1A**, STAR can output BAM files in both coordinate spaces, and thus, the effect of read mapping algorithm on our evaluation is eliminated. Although RSEM [22–23] is slower than some other algorithms, it estimates isoform abundance with overall high accuracy according to Kanitz et al. [28]. The featureCounts [26] and HTSeq [27] is comparable in terms of counting results, but featureCounts is considerably faster than HTSeq by an order of magnitude for gene-level summarization and requires far less computer memory. That’s why we choose RSEM (a transcript-based approach) and featureCounts (a ‘union exon’-based approach) in our evaluation.

To count reads mapped to individual genes in GENCODE database, the parameters for RSEM run is “-*p 7—seed 12345—quiet—time—no-bam-output—bam—paired-end—forward-prob = 0*”. In RSEM, a read is counted if and only if it 100% overlaps with an isoform. In contrast, featureCounts counts those reads that partially overlap with a gene exon as long as the overlap is adequately long. The overlap length threshold is set to 18 after discussion with the authors of featureCounts. It is noted that featureCounts does not assign reads to isoforms, and instead, it collapses all overlapping exons into ‘union exons’ first, and then summarizes the reads that intersect with ‘union exons’. If a read matches more than one feature, it is excluded from counting since there is no way to differentiate which one it is truly from. The parameters in featureCounts run were “*-p -T 4 -F GTF -a hg19*.*gencode*.*v19*.*gtf -t exon -g gene_id -s 2 -B -C—minReadOverlap 18*”. Only uniquely mapped reads are used in the counting step. Like the mapping step above, the counting metrics were collected and reported in **Table 1**.

### Gene quantification and differential analysis

A gene counts table and isoform counts table were generated by featureCounts and RSEM, respectively. RPKM is calculated by different approaches. For transcript-based approach, reads are first assigned to each transcript, and the corresponding RPKM for individual transcripts is calculated. Then the sum of transcript RPKM of the same gene represents the gene expression level. For ‘union exons’-based approach, the reads are assigned to gene directly, and the gene length is the total length of the ‘union exons’. With that, the gene RPKM is calculated according to RPKM definition.

The differential analysis was performed by R packages edgeR 3.10.2 and Limma/voom 3.22.10. We compared the UHRR with HBRR groups. All genes or isoforms with a fold change greater than 1.5 and a Benjamini-Hochberg adjusted p-value smaller than 0.05 were reported as differential expression genes or isoforms. The gene and isoform differential analysis results were further compared.

## Supporting Information

S1 Table Lists read counts table generated by FeatureCounts and RSEM. (XLSX)

S2 Table Lists the calculated RPKMs for the 4 samples using different approaches. (XLSX)
